# Evaluation of Biochemical, Haematological, and Histopathological Responses and Recovery Ability of Common Carp (*Cyprinus carpio* L.) after Acute Exposure to Atrazine Herbicide

**DOI:** 10.1155/2014/980948

**Published:** 2014-03-27

**Authors:** Jana Blahova, Helena Modra, Marie Sevcikova, Petr Marsalek, Lenka Zelnickova, Misa Skoric, Zdenka Svobodova

**Affiliations:** ^1^Department of Veterinary Public Health and Animal Welfare, University of Veterinary and Pharmaceutical Sciences Brno, Palackeho tr. 1/3, CZ61242 Brno, Czech Republic; ^2^Department of Pathological Morphology and Parasitology, University of Veterinary and Pharmaceutical Sciences Brno, Palackeho tr. 1/3, 612 42 Brno, Czech Republic

## Abstract

The aim of study was to evaluate the effect of atrazine exposure (5, 15, 20, and 30 mg*·*L^−1^) on common carp and the ability of regeneration. During 96 h exposure we observed abnormal behavior in fish exposed to 20 and 30 mg*·*L^−1^. Mortality and histological alterations were noticed only in the group exposed to 30 mg*·*L^−1^. Most experimental groups showed significantly (*P* < 0.05) lower values of haemoglobin, haematocrit, leukocyte, and lymphocyte and significantly higher values of monocytes, segmented and band neutrophile granulocytes, and also metamyelocytes and myelocytes. A significantly lower (*P* < 0.05) leukocyte count was also recorded in experimental groups (5 and 15 mg*·*L^−1^) after recovery period. Statistically significant (*P* < 0.05) alterations in glucose, total protein, lactate, phosphorus, calcium, and biopterin as well as in activities of ALT, AST, ALP, and LDH were found in most experimental groups. These changes were most apparent in the groups exposed to 20 and 30 mg*·*L^−1^. Most of the indices were found to be restored after the 7-day recovery period with the exception of LDH, ALT, and lactate in the group exposed to 15 mg*·*L^−1^. Our results showed that atrazine exposure had a profound negative influence on selected indices and also on histological changes of common carp.

## 1. Introduction

Atrazine is one type of chloro-*s*-triazine herbicides and it is by far most widely used in agriculture especially for the production of corn, sorghum, and sugarcane. They were developed specifically as a phytotoxin, which inhibits photosynthesis via competition with plastoquinone II at its binding site in the process of electron transport in photosystem II in target plants [1, 2].

Atrazine is considered moderately toxic to aquatic animals [2]. Deleterious effects of atrazine, which have been studied in different fish species, confirmed an induction of oxidative stress [3, 4], changes of behavior [5, 6], or biochemical alteration after acute, subchronic, or chronic exposure. Some studies investigated negative effects of atrazine on reproduction [7], immune response [8], or detoxifying system [9]. Exposure to atrazine could also cause histopathological changes in liver, kidney, gill, or other fish organs [6, 10]. In general, clinical symptoms such are alterations in fish behavior and pathological anatomical finding can be used as an indicator of acute poisoning [2].

In the European countries, the use of atrazine was completely prohibited in 2004 [11]. Despite its prohibition, atrazine is still frequently used by growers in more than 70 countries, with the top being the United States, Brazil, Argentina, Mexico, and China [1, 2]. Atrazine is listed as a priority substance in the European Water Framework. The annual average and the maximum allowable concentrations have been proposed for inland and other surface waters (0.6 and 2 *μ*g·L^−1^, resp.) [12].

Although the* s*-triazine ring makes atrazine molecule resistant, this herbicide undergoes physical, chemical, and biological degradation in the soil, as well as in the water column and in the sediment associated with water bodies [1]. The half-life of atrazine in soil, water, and sediments ranges from few weeks to more than 2 years depending on physicochemical properties of the environment. Atrazine may degrade into many metabolites, each of varying persistence and toxicity. The most common metabolites are hydroxyatrazine, deethylatrazine, deisopropylatrazine, didealkylatrazine, and deethylhydroxyatrazine [13]. Due to the persistence and mobility, atrazine and its degradation products are still detected in surface water and groundwater of countries, in which the application of atrazine was banned. According to the Czech Hydrometeorological Institute, water samples from Czech rivers in years 2005, 2006, 2007, and 2008 showed rates of occurrence of 88, 86, 46, and 60% in surface water, respectively, and the maximum concentrations in these years ranged from 0.3 to 1.0 *μ*g L^−1^. In the countries, where atrazine is still frequently used, the concentrations in streams, rivers, and lakes are severely higher. Obtained levels of atrazine in water are episodic with major peaks in spring and early summer after the field application in April and May [1, 3].

The aim of the present study was to investigate the toxic effect of atrazine on common carp (*Cyprinus carpio* L.) following 96 h exposure. Furthermore, the ability of organism regeneration was studied using 7-day recovery period (depuration in fresh water). The toxic effects were evaluated on the basis of results of haematological, biochemical, and histopathological examination of test organisms.

## 2. Materials and Methods

### 2.1. Design of Experiment

The total of 80 common carps (*C. carpio*) (mean body weight 88.4 ± 2.6 g) was obtained from a local hatchery (Pohořelice, PCL, Czech Republic) and used in the acute toxicity test with atrazine of 99.0% chemical purity (A2S, France). At first, the fish were randomly distributed into 8 aquarium tanks (*n* = 10) with dechlorinated water (volume of 100 L). After one week acclimatization to laboratory conditions, the fish were exposed to a range of atrazine concentrations (5, 15, 20, and 30 mg·L^−1^) for 96 h. In control and experimental groups with 5 and 15 mg·L^−1^ of atrazine we used 20 fish for each concentration; in the experimental groups with 20 and 30 mg·L^−1^ of atrazine we used only 10 fish for each concentration. The solution volumes were replaced once a day. The atrazine stock solutions were prepared in dimethyl sulfoxide with the final concentration of 0.005% (HPLC grade). Atrazine concentration was controlled by chromatographic analysis and did not decrease below 80% of the original concentration during the experiment. During the test, conditions of fish and water were checked at 24 h intervals and the number of dead fish was recorded for each concentration. The values of water quality were as follows: temperature 22 ± 1°C, oxygen saturation above 60%, and pH 7.6–8.1. After 96 h exposure, individual blood samples were taken from several fish in control (*n* = 12), 5 mg·L^−1^ (*n* = 12) and 15 mg·L^−1^ (*n* = 12) of atrazine groups and from all fish in 20 mg·L^−1^ (*n* = 10) a 30 mg·L^−1^ (*n* = 4) of atrazine experimental groups. Blood samples were obtained by a cardiac puncture and stabilized with 50 IU of heparin sodium salt per one mL of blood. The fish were killed by severing the spinal cord and selected organs (liver, gill, kidney, and skin) were taken for histopathological examination. The remaining fish in control (*n* = 8), 5 mg·L^−1^ (*n* = 8) and 15 mg·L^−1^ (*n* = 8) of atrazine groups was subjected to dechlorinated water for another 7 days to assess the recovery ability. At the end of the recovery period, individual blood samples and selected organs for histopathological examination were taken. Experimental procedures were in a compliance with the national legislation—Act No. 246/1992 Coll., on the Protection of Animals against Cruelty, as amended [14] and Decree No. 419/2012 Coll., on the Protection, Breeding, and use of Experimental Animals, as amended [15].

### 2.2. Haematological and Biochemical Indices of Blood Samples

The haematological indices were determined in heparinized blood and included erythrocyte count (RBC), haemoglobin concentration (Hb), haematocrit value (PCV), leukocyte count (WBC), and differential leukocyte count. This procedure was carried out using the unified method for haematological examination of fish [16]. Blood plasma, which was obtained from heparinized blood samples by centrifugation (4°C, 800 ×g, 10 min), was used for the determination of selected biochemical indices. Biochemical indices in blood plasma included glucose, total protein, albumin, alanine aminotransferase (ALT), aspartate aminotransferase (AST), alkaline phosphatase (ALP), lactate, lactate dehydrogenase (LDH), phosphorus, and calcium which were determined using biochemical analyzer Konelab 20i and commercial kits (Biovendor PCL, Czech Republic). Blood plasma was also used for the determination of biopterin and neopterin concentrations. The analysis of pterins was based on high performance liquid chromatography with fluorometric detection. For neopterin and biopterin analysis, 300 *μ*L of trichloroacetic acid (5%) was added to 300 *μ*L of standard or plasma. The samples were centrifuged at 800 ×g for 10 min at 20°C. The supernatant was filtered through a 0.45 *μ*m nylon filter and used for analysis. Elution was performed on a 150 × 4.6 mm, 5 *μ*m Zorbax Eclipse XBD-C18 column (Agilent Technologies, Santa Clara, CA). Isocratic elution was performed at a flow rate of 1 mL·min^−1^ with water/acetonitrile (96 : 4) at 35°C. Fluorescence detection at 353 nm and 438 nm for excitation and emission, respectively, was used to detect pterins selectively. The chromatographic analysis was accomplished by means of an Alliance 2695 chromatographic system (Waters, Milford, MA) with an FD 2475 fluorescent detector (Waters, Milford, MA). Neopterin, biopterin, and trichloroacetic acid were purchased from Sigma-Aldrich (St. Louis, MO). All solvents were of HPLC grade purity (Chromservis Ltd., CZ). The detection limits for neopterin and biopterin were 0.23 and 0.41 ng·mL^−1^, respectively. The limits of quantification for neopterin and biopterin were 1.85 and 2.45 ng·mL^−1^, respectively. The coefficient of variation was 3.8%.

### 2.3. Histopathological Examination

The selected fish tissues (liver, gill, cranial and caudal kidney, and skin) were prepared for histopathological examination, fixed in buffered 10% neutral formalin, dehydrated, embedded in paraffin wax, sectioned on a microtome at a thickness of 4 *μ*m, and stained with haematoxylin and eosin.

### 2.4. Determination of Atrazine in Water

Measurement of atrazine concentration in water samples was conducted using gas chromatography with ion trap tandem mass spectrometry. Sample preparation was based on simple liquid-liquid extraction into cyclohexane (1 : 1). The separation, identification, and quantification of atrazine were carried out using a Varian 450-GC gas chromatograph with 220-MS ion trap mass spectrometer and VF-5 ms (30 m × 0.25 mm) column (Varian, Inc., USA). A 1 *μ*L of aliquot of sample extract was injected in splitless mode. The injector temperature was 250°C. The initial oven temperature was set at 50°C for 1 min, increased in a rate of 30°C per min to 130°C for 1 min, increased in a rate of 16°C per min to 230°C, held for 1 min, increased in a rate of 60°C per min to 280°C, and held for 1 min. The total run time was 13.75 min. The certified standard of atrazine was purchased from Dr. Ehrenstorfer GmbH (Germany). All solvents were of GC/MS grade purity (Chromservis Ltd., CZ).

### 2.5. Statistical Analysis

Statistical assessment was carried out using Unistat 5.6 for Excel software (Czech Republic). Data were tested for normality (Shapiro-Wilk test) and a one-way analysis of variance (ANOVA) was applied to test the differences in measured indices among experimental groups for both tests, that is, for 96 h toxicity test and recovery test together. Individual differences among the means were tested successively using multiple comparison Tukey HSD test. The differential leukocyte count did not satisfy normal distribution; therefore a nonparametric Kruskal-Wallis test was applied. Significance was accepted at *P* < 0.05. All data are reported as mean ± standard error of mean (SEM).

## 3. Results

### 3.1. Mortality and Fish Behavior

**Figure 1:**
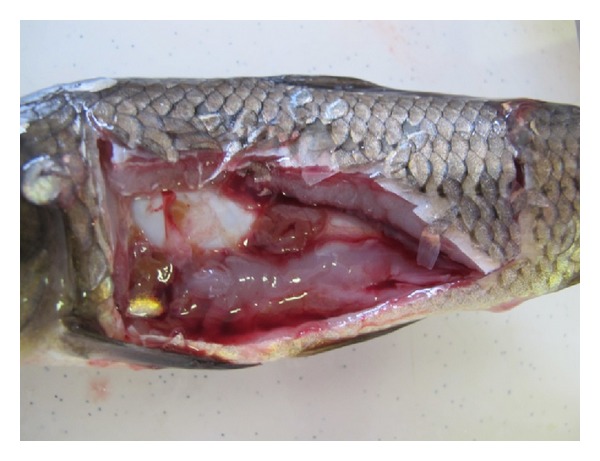
Experimental carp after 96 h exposure to 30 mg·L^−1^ of atrazine: transudate in the body cavity.

Changes in behavior were observed already after one hour of exposure to atrazine in the groups of fish in the highest concentrations (20 and 30 mg·L^−1^). The behavioral changes were also observed in the group exposed to 15 mg·L^−1^ after 24 hours, but these changes were not so intensive. Abnormal behavior included reduced reflexes, erratic swimming, loss of equilibrium, and accelerated respiration. In the highest concentration, the fish were lying on their side and were moving only in this position. No changes in behavior were observed in the control group and experimental group exposed to 5 mg·L^−1^ of atrazine as well as in all groups during the recovery period. Mortality was observed only in the highest concentration of atrazine (30 mg·L^−1^), in which the total mortality for 96 hours was 60%. In this group, fish began to die after 48 hours of atrazine exposure. Transudate in the body cavity and an increased injection of visceral vessels were found during autopsy in the fish exposed to the highest concentration of atrazine (30 mg·L^−1^) (Figure 1).

### 3.2. Haematological Profile of Blood

The results of haematological examination of blood samples from acute toxicity test and recovery period are given in Table 1. It is evident that the acute exposure to atrazine resulted in significant changes in almost all haematological indices, especially in the groups exposed to the highest concentrations of atrazine (20 and 30 mg·L^−1^). A nonsignificant decrease in erythrocyte count in the group exposed to 30 mg·L^−1^ subsequently led to a significant decrease (*P* < 0.05) in haemoglobin concentration and haematocrit value in this group compared to the control. A significant decrease (*P* < 0.05) in white blood cells was observed in all experimental groups exposed to atrazine for 96 h. Significantly lower (*P* < 0.05) leukocyte count was also recorded in experimental groups exposed to 5 and 15 mg·L^−1^ of atrazine after seven days of recovery period in pure water compared to the control group. Acute exposure to atrazine resulted in an intensive, significant (*P* < 0.05) alteration of differential leukocyte counts compared to the control group; no changes were noticed during the recovery period. A severalfold significant decrease (*P* < 0.05) in lymphocytes was found in experimental groups (15, 20, and 30 mg·L^−1^) during 96 h toxicity test. On the other hand, we obtained a severalfold significant increase (*P* < 0.05) in almost all experimental groups during 96 h atrazine exposure in the case of monocytes, segmented and band neutrophile granulocytes, metamyelocytes, and myelocytes.

### 3.3. Biochemical Profile of Blood Plasma

Results of blood plasma biochemical indices are shown in Table 2. After 96 h exposure, there were found significant (*P* < 0.05) alterations in the concentrations of glucose, total protein, albumin, lactate, phosphorus, and calcium as well as in the activities of ALT, AST, ALP. and LDH compared to the control group. Most of the biochemical parameters were found to be restored after a 7-day recovery period. Only in the case of LDH and ALT activities and the concentration of lactate, the indices in experimental group exposed to 15 mg·L^−1^ of atrazine for 96 h were significantly different (*P* < 0.05) even after the recovery period in comparison to the control group.

The results of pterins concentration in blood plasma are shown in Table 3. Because of an insufficient amount of blood samples taken from fish in experimental group exposed to 30 mg·L^−1^, the analysis of pterins was not performed in this concentration. Acute atrazine exposure resulted in an increase in biopterin content only in the experimental group exposed to 20 mg·L^−1^. No changes were found in neopterin concentration.

### 3.4. Histopathological Examination

**Figure 2:**
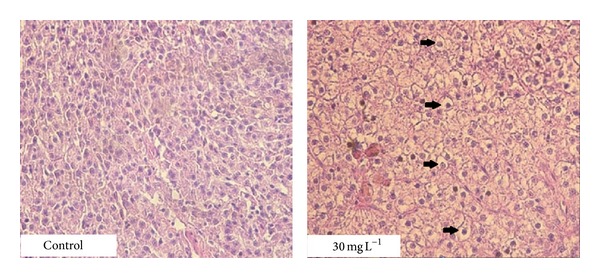
Results of histopathological examination of liver samples (arrows: diffuse hydropic to vacuolar degeneration of hepatocytes).

Histopathological examination revealed pathological lesions in fish only in the experimental group with the highest concentration of atrazine (30 mg·L^−1^). Morphological changes were observed in the liver and represented by moderate to marked dystrophic lesions of hepatocytes. There were morphological signs of the initial cell injury represented by hydropic to vacuolar degeneration of hepatocytes, dilatation of capillaries, and hyperaemia. Affected liver tissues were histopathologically compared with tissue sections from negative control group (Figure 2).

**Figure 3:**
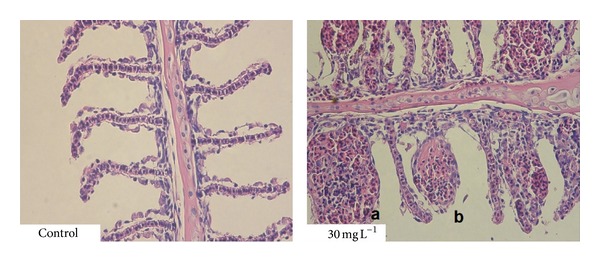
Results of histopathological examination of gill samples (a: severe lamellar teleangiectasis; dilated lamellar capillaries filled with erythrocytes; b: rupture of dilated lamellar capillary and pooling of the blood with formation of thrombi).

Marked lesions were also observed in the gill samples and represented by a severe multifocal lamellar teleangiectasis as a result of the rupture of the retaining pillar cells and dilation of the lamellar capillary and pooling of the blood with formation of thrombi. Affected tissues were histopathologically compared with tissue sections from negative control group (Figure 3). Tissues and organs of the fish in experimental groups exposed to atrazine at the concentrations of 5, 15, and 20 mg·L^−1^ exhibited no pathomorphological changes.

## 4. Discussion

Although the atrazine use has been banned in the Czech Republic and also in the European Union few years ago, the residues of this persistent compound are still found in the surface waters. The presented study showed that acute exposure to relatively high concentrations of atrazine could negatively affect the health status of common carp, which is connected with the changes in behavior, alteration in biochemical and haematological indices, and also pathological-anatomical findings in selected tissues of common carp.

In the highest concentrations (20 and 30 mg·L^−1^), the alterations in behavior were observed after one hour of atrazine exposure. Behavior changes were also registered in the experimental group exposed to 15 mg·L^−1^ of atrazine, but this abnormal behavior was observed after 24 hours of exposure. During the recovery period, no behavioral alterations were observed in the experimental groups. Behavior changes during exposure period were also reported by Chapadense et al. [17], who evaluated the toxic effects of atrazine on fish species tambaqui (*Colossoma macropomum*). They documented abnormal behavior such is lethargy, loss of equilibrium, increase in the frequency of opercular movements, and increase in the thickness of the inferior lips after 48 h of exposure to atrazine at the range of 20–25 mg·L^−1^. Moreover, they observed presence of hemorrhages in the eyes, lips, or even the whole body. Similar trends connected with uncoordinated behavior were also documented in freshwater fish* Channa punctatus* after 96 h exposure to sublethal concentrations of atrazine at the range of 4.2–10.6 mg·L^−1^ [18]. At the initial exposure, fish were alerted and stopped swimming; after some time they tried to avoid the toxic effects of atrazine with fast swimming and jumping and at the end of the experiment; fish lost their balance and became exhausted and lethargic. They also continuously secreted massive amounts of mucus from whole body continuously and the body pigmentation was decreased. Steinberg et al. [19] documented that exposure of zebrafish to atrazine caused changes in behavior, such as a significant preference of dark habitats. Surprisingly, this effect was already detected in the environmental concentration (5 *μ*g·L^−1^), in which 79% of the fish records were taken in the dark part.

Atrazine is rapidly metabolized in the liver and kidney and then excreted without any significant accumulation in the tissues of fish organism. Some studies also documented alterations of the gill tissues because of their direct contact with water, which allows entering the substance through them into the fish body [2, 13]. Therefore the exposure to atrazine in fish is most often associated with the degenerative changes in the kidney and gills and also with the alteration in the liver tissues [20–23]. Besides, atrazine has been proposed to exert adverse effects on the reproductive fitness of fish. This can lead to gonad abnormalities in both male and female fish [2, 10]. Fischer-Scherl et al. [20] documented alterations of different components of renal corpuscles and renal tubules in rainbow trout (*Oncorhynchus mykiss*) after exposure to atrazine at the range of 5–40 *μ*g·L^−1^ for 28 days. They also found necrosis of endothelial cells and renal hemopoietic tissue in experimental group exposed to the concentrations at the range of 80–2800 *μ*g·L^−1^. Similar results were obtained after exposure of rare minnow (*Gobiocypris rarus*) to atrazine for 28 days; histological observation revealed lesions in kidney tissues including an extensive expansion in the lumen, degenerative and necrotic changes of tubular epithelia, and shrinkage of the glomeruli at the concentration of 10 *μ*g·L^−1^. The same concentration also resulted in obvious lesions in the gill including hyperplasia, necrosis in epithelium region, aneurysm, and lamellar fusion [23]. Similarly, the gills changes were observed after acute 6 h exposure to atrazine in freshwater fish* Gnathonemus petersii* and they were represented by the breaks in the epithelium at 0.5 mg·L^−1^ and developed into deep pits at 5.0 mg·L^−1^ [24]. Steatosis in liver tissue was observable in grey mullet (*Liza ramanda*) after subchronic exposure to 170 *μ*g·L^−1^ of atrazine. But in fish exposed to atrazine and then returned to clean water, a reversal of the induced alterations and enhancement of the hepatic metabolism linked to detoxication mechanisms indicated hepatic recovery [22]. Mela et al. [4] studied the effects of acute atrazine exposure at the range of 2–100 *μ*g·L^−1^ in neotropical catfish (*Rhamdia quelen*) and histopathology examination of liver tissues revealed leukocyte infiltration, hepatocyte vacuolization like steatosis and necrosis areas, leading to raised lesion index levels in all tested concentrations. Surprisingly, the highest concentration of atrazine did not cause the alteration in kidney tissue in the current experiment, but we observed degenerative changes in liver tissue represented by marked hydropic to vacuolar degeneration of hepatocytes, dilatation of capillaries, and hyperaemia. Moreover, we obtained a severalfold significant increase in the activities of all liver enzymes such as ALT, AST, and ALP detected in plasma samples of all experimental groups and, also in the case of ALT, the significant increase in its activity recorded after the recovery period in the experimental group exposed to 15 mg·L^−1^ of atrazine. The other significant alterations represented by multifocal lamellar teleangiectasis were found in the gill tissues of fish from the experimental group exposed to 30 mg·L^−1^ of atrazine. The cause of the damage most likely results from the fact that atrazine denatures the epithelium of the gill filaments and this damage disrupts the ion regulatory function of the gills [13].

In our study, we also observed the formation of transudate in the body cavity; this finding was recorded only in the experimental group exposed to the 30 mg·L^−1^ of atrazine. We can assume that the presence of transudate in the body cavity can be connected with a decrease in oncotic pressure caused by hypoproteinaemia and hypoalbuminaemia, which were found in this experimental group. Similar results were documented in the toxicological studies with other triazine pesticides [25], in which the formation of transudate was also associated with the damage of epithelial cells of renal tubules [26].

In response to a stressor such as pesticide exposure, the fish undergo a series of biochemical and physiological changes in an attempt to compensate the challenge imposed on them and thus cope with the stress. Blood is one of the important pathophysiological reflectors of the whole body organism. Therefore, blood parameters such as haematological and biochemical indices can be used as important markers of diagnosing the structural and functional status of fish exposed to pesticides, which induce stress reaction [27, 28]. In our study, acute exposure to atrazine resulted in significant concentration-dependent changes in almost all haematological and biochemical indices and in the case of some indices these alterations were irreversible after 7-day depuration in atrazine-free water.

Leukocytes are involved in the regulation of immunological function and their alterations can be connected with immunotoxic potential of substances [2, 29]. Changes in leukocyte profile indicate the response of fish organism to stress reaction [30]. It appears that there are distinct differences in the sensitivity of fish in terms of their immune response to atrazine exposure [2]. In our study, the main haematological responses of common carp to the effects of acute exposure of atrazine were a significant decrease in white blood cells and lymphocytes and a significant increase in monocytes, segmented and band neutrophile granulocytes, and metamyelocytes and myelocytes counts. These changes were observed especially in the experimental groups exposed to the highest concentrations of atrazine and, only in the case of count of white blood cells, this alteration continued also during the recovery period. Declines in both leukocytes and lymphocytes counts indicate a reduction of nonspecific immunity and a stress condition of common carp after atrazine exposure. A decrease in leukocytes and lymphocytes was also reported by Velisek et al. [25, 26] after acute exposure to metribuzin in common carp and rainbow trout (*Oncorhynchus mykiss*).

In the erythrocyte profile, we observed alterations especially in the experimental group exposed to the highest concentration of atrazine. A significant decrease in haematocrit value and haemoglobin concentration can be interpreted as a compensatory response that reduces the oxygen carrying capacity to maintain gas exchange in the damaged gill lamellae. Our results are in line with those found by other authors, who assessed the effects of selected pesticides on haematological profile of fish blood [25, 26, 30–32].

Our results of blood plasma biochemical investigation indicated strong alterations in almost all indices as a stress response to atrazine exposure; the main biochemical responses were observed in glucose level and in the activities of liver enzymes. An increase in glucose levels detected in all experimental groups after 96 hours of atrazine exposure reflects an intense metabolic stress response of fish organism. The highest enhancement, which was more than dozen times, was observed in fish exposed to the highest concentration (30 mg·L^−1^). In spite of this, the glucose concentration was found to be restored to physiological levels after a 7-day recovery period. Similar trend was also observed in several studies, in which acute exposures of fish organisms to different pesticides were studied [25, 33].

In our experiment, we recorded significant elevations in the activities of all enzymes as conventional indicators of liver injury in fish exposed to different atrazine concentrations. The most pronounced alteration was found in ALT activity and the significantly increased activity of the enzyme was also observed after the recovery period. Alanine aminotransferase is predominantly present in hepatocytes and therefore its increased activity after the recovery period could reflect the damage of liver tissue [33]. Similar results were found by Neškovič et al. [21] who assessed effects of subacute (14 days) exposure to atrazine at the range of 1.5–6 mg·L^−1^ in common carp. In all analyses of blood serum, the activity of ALP demonstrated a significant increase ranging from 75 to 200% compared to the control group. On the other hand, the ALP activities in tissues (heart, liver, and kidney) showed significant decrease compared to the control group, which was associated with histopathological alteration found in these tissues.

Furthermore, we found a decline in the concentrations of plasma minerals (calcium and phosphorus), which is in close agreement with the findings of the study by Prasad and Reddy [34]. The authors observed significant changes of blood minerals in* Tilapia mossambicus* indicating disturbances in the hydromineral balance of the fish as a consequence of atrazine exposure. During the experiment, we recorded elevations of total protein and albumin especially in the experimental group exposed to 15 mg·L^−1^ of atrazine followed by the decline in these indices in experimental groups exposed to the highest concentrations. The protein concentration could be used as an indicator of general state of fish health and also as an indicator of stress [30].

Neopterin and biopterin belong to a group of unconjugated pterins and their levels in body fluids are often used as a biochemical marker of cell mediated immunity [35]. Unfortunately, there is only one study, which deals with the possibility to use the monitoring of pterins as potential markers of immune stress in fish organisms after pesticide exposure [36]. The authors studied the effects of subchronic exposure to prochloraz in common carp and they reported an increase in neopterin concentration, while biopterin concentration was not influenced. In the present study, we found an alteration only in the case of biopterin level in the experimental group exposed to 20 mg·L^−1^ after 96 hours. Our findings suggest an influencing of the fish immune system by acute atrazine exposure, which is further documented by changes in the count of white blood cells in the exposed groups.

## 5. Conclusion

In summary, this study gives further evidence that atrazine is toxic to fish organism and causes changes in biochemical and haematological indices and alteration of behavior and pathological anatomical finding of common carp. On the other hand, the results of the recovery test showed a high regeneration potential of organisms and confirmed that the responses of common carp were in most indices reversible when exposure was terminated. Irreversible changes were observed only in the activity of liver enzyme alanine aminotransferase and the count of white blood cells (especially lymphocytes), which confirms a hepatotoxic and immunosuppressive potential of atrazine.

## Figures and Tables

**Table 1:** Results of haematological examination during atrazine exposure and recovery period.

Indices	96 h toxicity test					7-day recovery test in pure water		
	Control	5 mg·L^−1^	15 mg·L^−1^	20 mg·L^−1^	30 mg·L^−1^	Control	5 mg·L^−1^	15 mg·L^−1^
RBC (T·L^−1^)	2.07 ± 0.12^ab^	1.55 ± 0.16^b^	2.21 ± 0.19^ab^	2.49 ± 0.10^a^	1.48 ± 0.46^b^	1.64 ± 0.06^b^	1.73 ± 0.17^b^	1.75 ± 0.12^ab^
Hb (g·L^−1^)	74.80 ± 1.35^a^	73.48 ± 1.88^a^	73.68 ± 3.63^a^	75.46 ± 2.95^a^	56.05 ± 7.19^b^	74.80 ± 1.35^a^	73.48 ± 1.88^a^	73.68 ± 3.63^a^
PCV (l·L^−1^)	0.24 ± 0.01^b^	0.24 ± 0.01^b^	0.22 ± 0.01^b^	0.23 ± 0.01^b^	0.18 ± 0.01^a^	0.23 ± 0.01^b^	0.25 ± 0.01^b^	0.23 ± 0.01^b^
WBC (G·L^−1^)	25.88 ± 1.73^a^	13.92 ± 1.36^b^	11.88 ± 1.05^b^	13.36 ± 1.34^b^	12.88 ± 4.30^b^	21.17 ± 1.06^a^	16.75 ± 2.25^b^	18.10 ± 1.80^b^
Lymphocytes (G·L^−1^)	7.18 ± 0.09^a^	5.99 ± 0.24^ab^	4.38 ± 0.31^b^	3.32 ± 0.33^b^	2.61 ± 0.83^b^	6.73 ± 0.17^a^	6.66 ± 0.13^a^	7.12 ± 0.09^a^
Monocytes (G·L^−1^)	0.08 ± 0.02^a^	0.08 ± 0.02^a^	0.14 ± 0.04^a^	0.12 ± 0.04^a^	0.30 ± 0.09^b^	0.09 ± 0.03^a^	0.10 ± 0.03^a^	0.10 ± 0.03^a^
NG-Segment (G·L^−1^)	0.12 ± 0.05^a^	0.43 ± 0.12^b^	0.52 ± 0.10^b^	0.48 ± 0.15^b^	0.51 ± 0.18^b^	0.16 ± 0.05^a^	0.21 ± 0.04^a^	0.18 ± 0.05^a^
NG-Bands (G·L^−1^)	0.08 ± 0.02^a^	0.26 ± 0.05^a^	0.72 ± 0.11^b^	0.92 ± 0.12^b^	1.46 ± 0.43^b^	0.08 ± 0.05^a^	0.12 ± 0.04^a^	0.07 ± 0.02^a^
Metamyelocytes (G·L^−1^)	0.11 ± 0.04^a^	0.64 ± 0.10^b^	1.58 ± 0.20^c^	1.94 ± 0.24^c^	1.83 ± 0.23^c^	0.26 ± 0.08^a^	0.32 ± 0.09^a^	0.19 ± 0.03^a^
Myelocytes (G·L^−1^)	0.09 ± 0.02^a^	0.18 ± 0.06^a^	0.37 ± 0.07^b^	0.79 ± 0.15^b^	0.85 ± 0.33^b^	0.07 ± 0.02^a^	0.09 ± 0.03^a^	0.05 ± 0.02^a^

Data are expressed as mean ± standard error of mean. Significant differences (*P* < 0.05) are indicated by different alphabetic superscripts (a legend: RBC: erythrocyte count; Hb: haemoglobin concentration; PCV: haematocrit value; WBC: leukocyte count; NG: neutrophile granulocytes).

**Table 2:** Results of biochemial examination during atrazine exposure and recovery period.

Indices	96 h toxicity test					7-day recovery test in pure water		
	Control	5 mg·L^−1^	15 mg·L^−1^	20 mg·L^−1^	30 mg·L^−1^	Control	5 mg·L^−1^	15 mg·L^−1^
Glucose (mmol·L^−1^)	3.42 ± 0.20^a^	9.15 ± 0.62^b^	23.03 ± 1.30^c^	22.47 ± 2.32^c^	28.02 ± 3.79^c^	3.55 ± 0.24^a^	3.55 ± 0.26^a^	3.39 ± 0.23^a^
Total protein (g·L^−1^)	19.59 ± 1.11^a^	20.97 ± 0.91^a^	26.52 ± 0.91^b^	23.12 ± 1.34^ab^	17.13 ± 4.7^a^	22.40 ± 0.62^ab^	19.36 ± 0.40^a^	21.72 ± 0.47^ab^
Albumin (g·L^−1^)	6.02 ± 0.39^ab^	6.99 ± 0.53^bc^	8.05 ± 0.29^b^	3.87 ± 0.70^a^	4.59 ± 2.12^ac^	5.93 ± 0.40^ab^	5.65 ± 0.25^ac^	6.20 ± 0.32^bc^
ALT (*μ*katal·L^−1^)	0.17 ± 0.01^a^	0.27 ± 0.03^acd^	0.46 ± 0.09^bc^	0.50 ± 0.07^bd^	0.60 ± 0.15^b^	0.25 ± 0.01^ac^	0.26 ± 0.02^ac^	0.49 ± 0.06^bd^
AST (*μ*katal·L^−1^)	2.33 ± 0.13^a^	2.45 ± 0.38^a^	3.33 ± 0.31^ac^	5.15 ± 0.36^b^	4.73 ± 0.67^bc^	3.31 ± 0.33^ac^	3.27 ± 0.14^ac^	2.81 ± 0.27^a^
ALP (*μ*katal·L^−1^)	0.21 ± 0.03^a^	0.32 ± 0.05^ab^	0.42 ± 0.02^b^	0.37 ± 0.03^bc^	0.24 ± 0.07^ac^	0.30 ± 0.02^ab^	0.30 ± 0.03^ab^	0.35 ± 0.03^bc^
LDH (*μ*katal·L^−1^)	3.98 ± 0.58^ab^	3.02 ± 0.61^ab^	4.57 ± 1.13^ab^	10.15 ± 0.89^c^	5.84 ± 1.35^b^	5.26 ± 0.65^b^	2.22 ± 0.13^a^	2.02 ± 0.21^a^
Lactate (mmol·L^−1^)	1.64 ± 0.30^b^	0.73 ± 0.10^ad^	0.90 ± 0.10^ab^	0.94 ± 0.10^ab^	0.40 ± 0.10^a^	1.45 ± 0.20^bd^	1.62 ± 0.13^b^	2.63 ± 0.20^c^
Phosphorus (mmol·L^−1^)	2.07 ± 0.08^c^	1.28 ± 0.11^ad^	1.12 ± 0.06^a^	1.55 ± 0.08^bd^	1.07 ± 0.10^a^	1.76 ± 0.09^bc^	1.62 ± 0.10^bd^	1.88 ± 0.07^bc^
Calcium (mmol·L^−1^)	2.04 ± 0.02^bc^	2.06 ± 0.03^c^	1.86 ± 0.03^b^	1.52 ± 0.03^a^	1.51 ± 0.03^a^	1.90 ± 0.08^bc^	1.91 ± 0.02^bc^	2.03 ± 0.03^bc^

Data are expressed as mean ± standard error of mean. Significant differences (*P* < 0.05) are indicated by different alphabetic superscripts (a legend: ALT: alanine aminotransferase; AST: aspartate aminotransferase; ALP: alkaline phosphatase; LDH: lactate dehydrogenase).

**Table 3:** Results (mean ± standard error of mean) of pterins in blood plasma during atrazine exposure and recovery period.

	Neopterin (mmol·L^−1^)	Biopterin (mmol·L^−1^)
	96 h toxicity test	
Control	25.61 ± 1.36^a^	47.53 ± 3.56^ac^
5 mg·L^−1^	27.99 ± 2.81^a^	49.29 ± 4.40^a^
15 mg·L^−1^	19.09 ± 1.48^a^	49.32 ± 5.71^ac^
20 mg·L^−1^	27.22 ± 3.66^a^	74.90 ± 6.24^b^

	7-day recovery test in pure water	
Control	24.18 ± 1.89^a^	36.60 ± 3.71^ac^
5 mg·L^−1^	21.69 ± 1.12^a^	31.56 ± 2.62^c^
15 mg·L^−1^	22.91 ± 1.63^a^	34.97 ± 4.34^ac^

Significant differences (*P* < 0.05) are indicated by different alphabetic superscripts.

## References

[1] LeBaron HM, McFarland JE, Burnside OC (2008). *The Triazines Herbicides 50 Years Revolutionizing Agriculture*.

[2] Solomon KR, Carr JA, Du Preez LH (2008). Effects of atrazine on fish, amphibians, and aquatic reptiles: a critical review. *Critical Reviews in Toxicology*.

[3] Blahová J, Plhalová L, Hostovský M (2013). Oxidative stress responses in zebrafish *Danio rerio* after subchronic exposure to atrazine. *Food and Chemical Toxicology*.

[4] Mela M, Guiloski IC, Doria HB (2013). Effects of herbicide atrazine in neotropical catfish (*Rhamdia quelen*). *Ecotoxicology and Environmental Safety*.

[5] Saglio P, Trijasse S (1998). Behavioral responses to atrazine and diuron in goldfish. *Archives of Environmental Contamination and Toxicology*.

[6] Plhalova L, Blahova J, Mikuliková I (2012). Effects of subchronic exposure to atrazine on zebrafish (*Danio rerio*). *Polish Journal of Veterinary Science*.

[7] Tillitt DE, Papoulias DM, Whyte JJ, Richter CA (2010). Atrazine reduces reproduction in fathead minnow (*Pimephales promelas*). *Aquatic Toxicology*.

[8] Kreutz LC, Barcellos LJG, dos Santos ED, Pivato M, Zanatta R (2012). Innate immune response of silver catfish (*Rhamdia quelen*) exposed to atrazine. *Fish and Shellfish Immunology*.

[9] Fu Y, Li M, Liu C (2013). Effect of atrazine and chlorphyrofos exposure on cytochrome P450 contents and enzyme activities in common carp gills. *Ecotoxicology and Environmental Safety*.

[10] Paulino MG, Souza NES, Fernandes MN (2012). Subchronic exposure to atrazine induces biochemical and histopathological changes in the gills of a neotropical freshwater fish, *Prochilodus lineatus*. *Ecotoxicology and Environmental Safety*.

[11] European Commission COMMISSION DECISION of 10 March 2004 concerning the non-inclusion of atrazine in Annex I to Council Directive 91/414/EEC and the withdrawal of authorisations for plant protection products containing this active substance (No. 2004/248/EC).

[12] European Commission Directive 2008/105/EC of the European Parliament and of the Council of 16 December 2008 on environmental quality standards in the field of water hggh83/513/EEC, 84/156/EEC, 84/491/EEC, 86/280/EEC and amending Directive 2000/60/EC of the European Parliament and of the Council.

[13] Graymore M, Stagnitti F, Allinson G (2001). Impacts of atrazine in aquatic ecosystems. *Environment International*.

[16] Svobodová Z, Pravda D, Modrá H *Methods of Haematological Examination of Fish*.

[17] Chapadense PFG, Castro FDJ, Almeida JA, Moron SE (2009). Toxicity of atrazine herbicide in *Colossoma macropomum*. *Revista Brasileira de Saúde e Produção Animal*.

[18] Nwani CD, Lakra WS, Nagpure NS, Kumar R, Kushwaha B, Kumar SK (2010). Toxicity of the herbicide atrazine: effects on lipid peroxidation and activities of antioxidant enzymes in the freshwater fish *Channa punctatus* (Bloch). *International Journal of Environmental Research and Public Health*.

[19] Steinberg CEW, Lorenz R, Spieser OH (1995). Effects of atrazine on swimming behavior of zebrafish, *Brachydanio rerio*. *Water Research*.

[20] Fischer-Scherl T, Veeser A, Hoffmann RW, Kuhnhauser C, Negele R, Ewringmann T (1991). Morphological effects of acute and chronic atrazine exposure in rainbow trout (*Oncorhynchus mykiss*). *Archives of Environmental Contamination and Toxicology*.

[21] Neškovič NK, Elezovič I, Karan V, Pokeksič V, Budimir M (1993). Acute and subacute toxicity of atrazine to carp (*Cyprinus carpio* L.). *Ecotoxicology and Environmental Safety*.

[22] Biagianti-Risbourg S, Bastide J (1995). Hepatic perturbations induced by a herbicide (atrazine) in juvenile grey mullet *Liza ramada* (Mugilidae, Teleostei): an ultrastructural study. *Aquatic Toxicology*.

[23] Yang L, Zha J, Li W, Li Z, Wang Z (2010). Atrazine affects kidney and adrenal hormones (AHs) related genes expressions of rare minnow (*Gobiocypris rarus*). *Aquatic Toxicology*.

[24] Alazemi BM, Lewis JW, Andrews EB (1996). Gill damage in the freshwater fish *Gnathonemus petersii* (family: Mormyridae) exposed to selected pollutants: an ultrastructural study. *Environmental Technology*.

[25] Velisek J, Svobodova Z, Piackova V, Sudova E (2009). Effects of acute exposure to metribuzin on some hematological, biochemical and histopathological parameters of common carp (*Cyprinus carpio* L.). *Bulletin of Environmental Contamination and Toxicology*.

[26] Velisek J, Svobodova Z, Piackova V (2008). Effects of metribuzin on rainbow trout (*Oncorhynchus mykiss*). *Veterinarni Medicina*.

[27] Adhikari S, Sarkar B, Chatterjee A, Mahapatra CT, Ayyappan S (2004). Effects of cypermethrin and carbofuran on certain hematological parameters and prediction of their recovery in a freshwater teleost, *Labeo rohita* (Hamilton). *Ecotoxicology and Environmental Safety*.

[28] Evans DH, Claiborne JB (2005). *The Physiology of Fishes*.

[29] Evans GO (2009). *Animal Hematotoxicology—A Practical Guide for Toxicologists and Biomedical Researchers*.

[30] Ramesh M, Srinivasan R, Saravana M (2009). Effect of atrazine (Herbicide) on blood parameters of common carp *Cyprinus carpio* (Actinopterygii: Cypriniformes). *African Journal of Environmental Science and Technology*.

[31] Prasad TAV, Srinivas T, Rafi GM, Reddy DC (1991). Effect in vivo of atrazine on haematology and O_2_ consumption in fish, Tilapia mossambica. *Biochemistry International*.

[32] Jee JH, Masroor F, Kang JC (2005). Responses of cypermethrin-induced stress in haematological parameters of Korean rockfish, *Sebastes schlegeli* (Hilgendorf). *Aquaculture Research*.

[33] Mikulikova I, Modra H, Blahova J (2013). Recovery ability of common carp (*Cyprinus carpio*) after a short-term exposure to terbuthylazine. *Polish Journal of Veterinary Science*.

[34] Prasad TAV, Reddy DC (1994). Atrazine toxicity on hydromineral balance of fish, *Tilapia mossambicus*. *Ecotoxicology and Environmental Safety*.

[35] Hoffmann G, Wirleitner B, Fuchs D (2003). Potential role of immune system activation-associated production of neopterin derivatives in humans. *Inflammation Research*.

[36] Maršálek P, Mikulíková I, Modrá H, Svobodová Z Effect of prochloraz fungicide on neopterin and biopterin levels in blood plasma of common carp.

